# Functional Roles of SPINK1 in Cancers

**DOI:** 10.3390/ijms22083814

**Published:** 2021-04-07

**Authors:** Tsung-Chieh Lin

**Affiliations:** Genomic Medicine Core Laboratory, Department of Medical Research and Development, Chang Gung Memorial Hospital, Linkou 333, Taoyuan City, Taiwan; tclin1980@cgmh.org.tw; Tel.: +886-3-3281-200 (ext. 7722)

**Keywords:** SPINK1, prognosis, carcinogenesis

## Abstract

Serine Peptidase Inhibitor Kazal Type 1 (SPINK1) is a secreted protein known as a protease inhibitor of trypsin in the pancreas. However, emerging evidence shows its function in promoting cancer progression in various types of cancer. SPINK1 modulated tumor malignancies and induced the activation of the downstream signaling of epidermal growth factor receptor (EGFR) in cancer cells, due to the structural similarity with epidermal growth factor (EGF). The discoverable SPINK1 somatic mutations, expressional signatures, and prognostic significances in various types of cancer have attracted attention as a cancer biomarker in clinical applications. Emerging findings further clarify the direct and indirect biological effects of SPINK1 in regulating cancer proliferation, metastasis, drug resistance, transdifferentiation, and cancer stemness, warranting the exploration of the SPINK1-mediated molecular mechanism to identify a therapeutic strategy. In this review article, we first integrate the transcriptomic data of different types of cancer with clinical information and recent findings of SPINK1-mediated malignant phenotypes. In addition, a comprehensive summary of SPINK1 expression in a pan-cancer panel and individual cell types of specific organs at the single-cell level is presented to indicate the potential sites of tumorigenesis, which has not yet been reported. This review aims to shed light on the roles of SPINK1 in cancer and provide guidance and potential directions for scientists in this field.

## 1. Introduction

**Figure 1:**
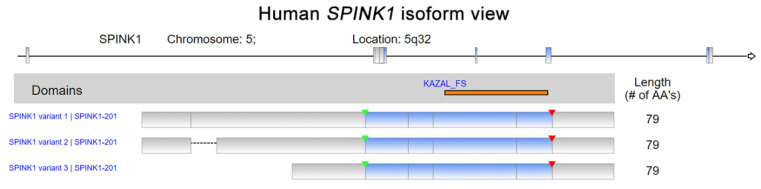
Human Serine peptidase inhibitor Kazal type I (*SPINK1*) isoform view. The data were retrieved and analyzed from Refseq. The matching protein domains in various RNA isoforms are marked and located in orange. The start of transcription and position of a stop codon are indicated by green and red arrowheads, respectively.

Serine peptidase inhibitor Kazal type I (SPINK1) was first discovered in bovine pancreas extracts by Kazal et al., and the molecule was designated a pancreatic secretory trypsin inhibitor (PSTI) [1]. SPINK1, which is also known as tumor-associated trypsin inhibitor (TATI), was further isolated from the urine of ovarian cancer patients by another research group [2]. SPINK1 and TATI were later characterized as identical molecules [3]. SPINK1 was first demonstrated to be released by acinar cells in the exocrine pancreas into the pancreatic duct and to interact with trypsin, inhibiting its activity both intracellularly and extracellularly [1]. The human *SPINK1* gene encodes an mRNA that is spliced into three transcript variants, which can be translated into a 79-amino acid peptide, including a 23-amino-acid signal peptide (Figure 1) [4,5]. In addition, the in silico prediction and precise splicing outcomes of the pathogenic *SPINK1* intronic variants have been reported [6]. Emerging evidence has demonstrated the relationship between SPINK1 and cancer progression [7,8]. SPINK1, as a senescence-associated secretory phenotype (SASP) factor, is produced in human stromal cells after genotoxic treatment because of DNA damage elicited by NF-κB and C/EBP signaling activation [9]. Interestingly, a SPINK1 interaction with epidermal growth factor receptor (EGFR) in rat liver hepatocyte line of BRL-3A in vitro has been indicated [10]. Additionally, an immunoreactivity study of colorectal cancer showed a positive Pearson’s correlation between SPINK1 and EGFR intensity [11]. Moreover, SPINK1 reprograms the expression profile of prostate cancer cells, leading to prominent epithelial–endothelial transition (EET), a phenotypic switch mediated by EGFR signaling [9]. Hence, the interaction of SPINK1 with EGFR has attracted attention for its potentially pivotal biological functions, especially in modulating cancer progression. Functionally, the SPINK1-driven biological effect induced by EGFR signaling was reported in ovarian cells [12]. In pancreatic cancer cell lines, SPINK1 was coprecipitated with EGFR in an immunoprecipitation experiment and trigger cancer cell proliferation via activating EGFR downstream signaling [13]. In prostate cancer, EGFR was found to mediate SPINK1′s biological function when triggering the epithelial–mesenchymal transition [14].

Current evidence emphasizes the urgent need to clarify the prognostic value of SPINK1 and unravel the SPINK1-dependent molecular mechanisms involved in human cancers. In this review article, SPINK1 expression is shown on a single-cell scale in various tissues. Previous review articles have illustrated SPINK1′s biological functions [7,8,15]. Here, we further review and summarize the recently published data focusing on integrated evidence that illustrates SPINK1 expression levels in a broad range of cancer types together with SPINK1-dependent biological effects on the regulation of several critical factors in processes related to cancer progression, including cancer proliferation, metastasis, drug resistance, cancer stemness, and transdifferentiation. In addition to the biological function of SPINK1 in cancer, its clinical and prognostic significance in multiple cancer types is demonstrated.

## 2. SPINK1 Mutations and Cancer

Understanding the mutational signature of *SPINK1* will improve the development and management of the step-up approach for cancer patients, specifically those harboring aberrant *SPINK1*. Recently, a novel technique, the immunocapture-liquid chromatography-mass spectrometry (IC-LC-MS) assay, was validated as able to detect and quantify serum SPINK1, including mutant forms N34S (SPINK1) and P55S (SPINK1) [16]. SPINK1 protects against the premature activation of trypsinogen and progression of acute pancreatitis, which may lead to carcinogenesis [17,18]. Previous studies have claimed a correlation of *SPINK1* mutations with a higher risk for pancreatic cancer, particularly in patients with chronic pancreatitis [19]. Patients with chronic pancreatitis (CP) due to the *SPINK1* gene mutation c.101A>G (*p*. N34S) detected by pyrosequencing were reported to have a 12-fold higher risk of developing pancreatic cancer than controls (Cox HR: 12.0 (3.0–47.8); *p* < 0.001) [20,21]. However, another cohort from Finland enrolling 188 patients with pancreatic malignant tumors showed that the N34S mutation was present in only seven cases (3.7%). The frequency of the N34S mutation in healthy controls was significantly higher than that reported in other countries [22]. A study using two pancreatic adenocarcinoma cell lines, PaCa44 and PancTu-I, harboring the heterozygous N34S variant further showed reduced SPINK1 levels compared with wild-type SPINK1. The negative regulation was due to the c.−4141G>T variant consistently found in the cells [23]. In addition to the frequently reported N34S mutation in the West, a retrospective study in China indicated that the c.194+2T>C mutation of *SPINK1* was present in 44.9% of patients with idiopathic chronic pancreatitis [24]. In particular, the metastatic pancreatic ductal adenocarcinoma patients with chronic pancreatitis were found to harbor the c.194+2T>C mutation [25]. The aforementioned studies showed the potential association of *SPINK1* mutations with the risk of cancer. However, whether those mutant forms of *SPINK1* are independent factors of cancer remains to be explored.

## 3. *SPINK1* Expression in Cancers

**Figure 2:**
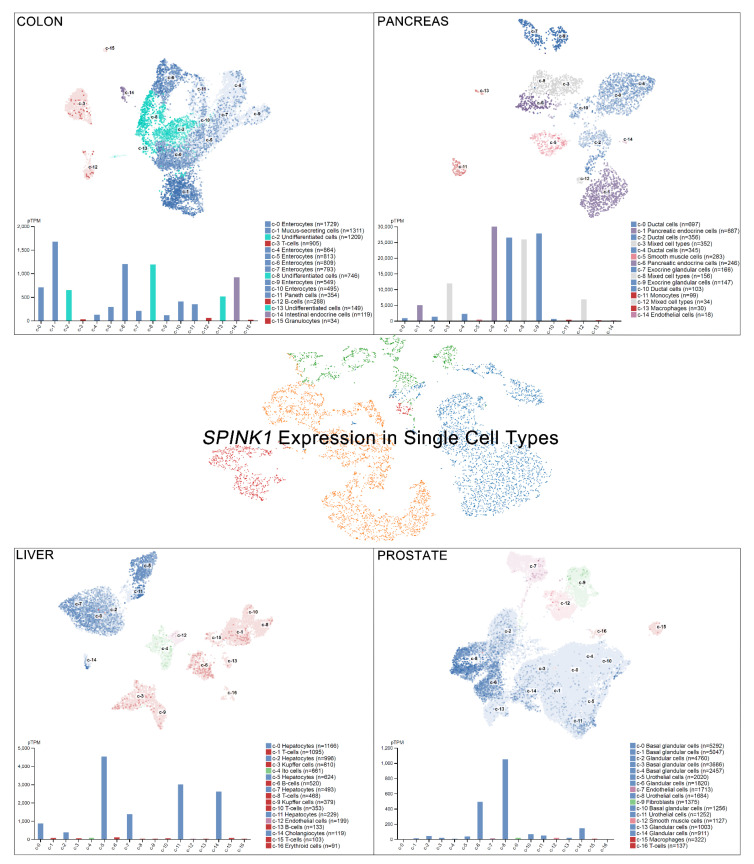
SPINK1 expression in single-cell types. The SPINK1 expression level was analyzed by single-cell RNA sequencing (scRNA-seq) using different human tissues (The Human Protein Atlas_ https://www.proteinatlas.org/, accessed on January 2021). RNA expression in the single-cell-type clusters identified in each tissue was visualized by a UMAP (Uniform Manifold Approximation and Projection) plot (top) and a bar chart (bottom). The read counts were normalized to transcripts per million protein-coding genes (pTPM) for each of the single-cell clusters.

Single-cell RNA sequencing (scRNA-seq) has become a powerful tool to delineate the composition of different cell types or states in a given tissue, determined by differentially expressed gene sets [26,27,28,29]. Recently, scRNA-seq of normal tissue led to the discovery of multiple cell types contributing to cancer [30]. A new single-cell-type atlas with publicly available genome-wide expression scRNA-seq data of 192 individual cell-type clusters from 13 different human tissues was launched in November 2020 (The Human Protein Atlas, accessed on January 2021) [31]. The relative SPINK1 expression in the top four tissues displaying high *SPINK1* levels, including the colon, prostate, liver, and pancreas, is shown on a single-cell scale (Figure 2). Relatively high SPINK1 expression was detected in enterocytes, mucus-secreting cells, intestinal endocrine cells, and undifferentiated cells of colon tissue. Pancreatic endocrine cells, exocrine glandular cells, and mixed cell types in the pancreas all showed SPINK1 expression. In addition, SPINK1 was specifically detected in hepatocytes and cholangiocytes compared with other cell types in liver tissue. In the prostate, SPINK1 was found in glandular cells and urothelial cells. The observation further suggests the potential sites of SPINK1 for playing roles in tumorigenesis. Importantly, data of immunohistochemical investigations were released (The Human Protein Atlas, https://www.proteinatlas.org/, accessed on January 2021) [31]. SPINK1 was relatively highly detected in tissues, including the stomach, duodenum, small intestine, colon, rectum, pancreas, and urinary bladder that is consistent with results found in colon and pancreas after comparing with single-cell RNA sequencing data. Actually, in colorectal cancer, a high percentage of positive immunohistochemical staining of SPINK1 was observed in cancer patients [32,33], suggesting a potential cause of tumorigenesis by abnormal SPINK1 overexpression.

**Figure 3:**
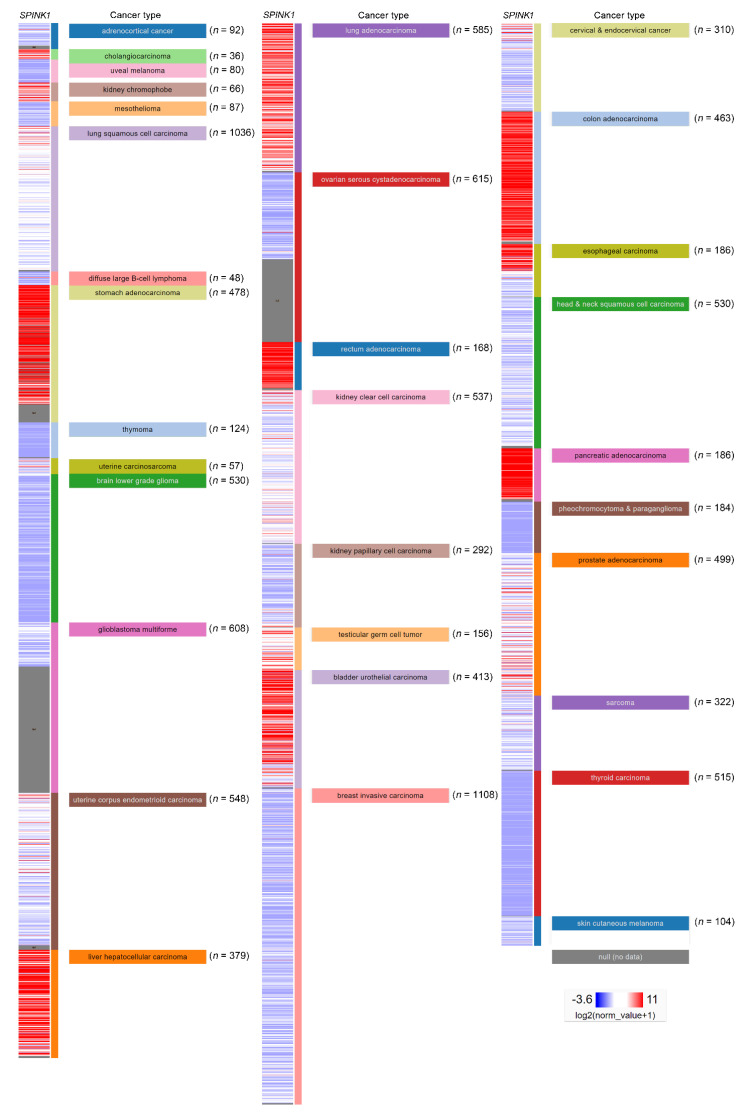
*SPINK1* expression view in a pan-cancer panel. In a pan-cancer dataset, *SPINK1* expression levels were presented separately for 32 cancer types. The red color in the heat map represents high *SPINK1* expression. The blue color in the heat map represents low *SPINK1* expression. The raw data were retrieved from The Cancer Genome Atlas (TCGA) database.

In addition, the regulatory mechanism of SPINK1 expression in cancer types has been reported. The *SPINK1* gene contains an IL-6 responsive element. A connection was observed in the colorectal cancer cell lines Colo205 and HT-29, in which the SPINK1 level was increased by both fibroblast-derived and recombinant IL-6 treatment [34]. Furthermore, IL-6 autocrine signaling was reported in an ovarian clear cell carcinoma study that IL-6 could regulate SPINK1 expression [35]. Clinically, another study using immunohistochemical staining detected the SPINK1 level in a high percentage of colorectal cancer patients [33]. In a prostate cancer model, miR-338-5p/miR-421 was epigenetically silenced in SPINK1-positive prostate cancer, and miR-338-5p/miR-421 was characterized as post-transcriptionally regulating *SPINK1* via 3′UTR binding [36]. The androgen receptor and corepressor REST have also been characterized as transcriptional repressors of *SPINK1*, while antagonists of the androgen receptors that alleviate this repression have been discovered [37]. Interestingly, SPINK1 modulates the tumor microenvironment, and its expression was specifically detected in the stromal cells of prostate cancer patients after chemotherapy [9]. In localized prostate cancer, SPINK1 was found exclusively absent in patients with homozygous PTEN deletion or ERG expressions [38,39]. In addition, the mutual exclusivity of SPINK1 expression and ETS fusion status had been reported in prostate cancer [40]. In liver cancer, a low SPINK1 expression score was found in cirrhosis patients compared with that in well-differentiated hepatocellular carcinoma (WD-HCC) patients. In addition, a significant difference in SPINK1 expression between WD-HCC and high-grade dysplastic nodules (HGDNs) was observed, suggesting a diagnostic role for SPINK1 in hepatocellular carcinoma [41]. The SPINK1 expression level in another hepatocellular carcinoma cohort was investigated using a tissue assay of 273 paired tumor and paratumor tissues. The SPINK1 level was significantly higher in the tumor tissues (*p* < 0.001) and correlated with portal vein tumor thrombus formation (*p* < 0.019) [42]. SPINK1 may also play a pivotal role in early hepatocellular carcinoma development because the investigation showed significant demethylation of SPINK1 in early hepatocellular carcinoma compared with HGDNs. The study further indicated that SPINK1 expression may be due to ER stress-induced SPINK1 demethylation during liver cancer progression [43]. Furthermore, SPINK1 was overexpressed in up to 70% of human hepatocellular carcinomas, and its expression level exhibited a positive correlation with CDH17 [44]. SPINK1 was highly expressed in non-small cell lung cancer compared with adjacent normal tissue samples [45]. A similar result was verified in a cell line panel study showing relatively high SPINK1 protein and RNA expression levels in the H460, H1299 and A549 lung cancer cell lines compared with those in normal human bronchial epithelial (HBE) cells [45]. A comprehensive project investigating the combination of multicancer transcriptomic data with matched clinical information was released by the University of California, Santa Cruz (*n* = 12,839) [46]. These transcriptomic data were mainly obtained after performing microarray experiments and RNA sequencing (RNA-Seq) on a pan-cancer scale, and the raw data were retrieved from the public database The Cancer Genome Atlas (TCGA), showing relative *SPINK1* expression after normalization in various types of cancer (Figure 3). *SPINK1* was relatively highly expressed in cholangiocarcinoma, kidney chromophobe cancer, stomach adenocarcinoma and liver hepatocellular carcinoma, lung adenocarcinoma, rectum adenocarcinoma, testicular germ cell tumor, urothelial bladder carcinoma, colon adenocarcinoma, and pancreatic adenocarcinoma. Additionally, lower *SPINK1* levels were observed in uveal melanoma, mesothelioma, diffuse large B-cell lymphoma, thymoma, brain lower grade glioma, ovarian serous cystadenocarcinoma, kidney papillary cell carcinoma, breast invasive carcinoma, head and neck squamous cell carcinoma, pheochromocytoma and paraganglioma, thyroid carcinoma and skin cutaneous melanoma.

## 4. Correlation with the Clinical Outcome

Studies have evaluated the prognostic significance of SPINK1 in cancer. SPINK1 was characterized as a prognostic marker for lung adenocarcinoma [47,48]. Lung adenocarcinoma patients harboring higher SPINK1 levels were associated with unfavorable overall survival and progression-free survival [45,48]. The association with poor overall survival was also observed in a hepatocellular carcinoma cohort of 273 cases (*p* = 0.029) [42]. A correlation with poor survival was discovered in the non-serous, histological tumor subtypes of ovarian cancer, including the endometrioid, clear cell, and mucinous subtypes [12]. SPINK1 is also considered one of nine key biomarkers to predict overall survival in patients harboring muscle-invasive bladder cancer (MIBC) [49]. Additionally, the serum SPINK1 level demonstrates its prognostic value, and a negative correlation of SPINK1 with disease-free survival was observed in prostate cancer [2]. SPINK1 overexpression was also positively correlated with prostate cancer-specific mortality in patients with biochemical and clinical recurrence after prostatectomy [50], and the relationship between SPINK1 and biochemical recurrence after surgical resection was exclusively shown in patients with aggressive subtypes of ETS-negative prostate cancers [45]. SPINK1 was not a predictor of mortality or overall survival among prostate cancer patients who had undergone radical prostatectomy. However, a prognostic value in patients with metastasis was observed [51]. Similar results were reported with Japanese men, for whom SPINK1 expression was not correlated with overall survival. Nevertheless, a significant association between the SPINK1 expression status and a shorter time to castration resistance (CRPC) was reported [52]. Patients with high SPINK1 protein levels showed an aggressive clinical course in ductal adenocarcinoma compared with those with acinar prostatic adenocarcinoma [53]. However, another report indicated a lack of association with pathologic or oncologic outcomes in patients undergoing radical prostatectomy in African-American (AA) men and European Americans (EA) [54]. A study enrolling 155 biopsy specimens from initially diagnosed bone metastatic prostate cancer showed a significant association of SPINK1 with the occurrence of castration-resistant prostate cancer (CRPC). The results further indicated that SPINK1 is an independent prognostic factor whose level is correlated with the adverse CRPC-free survival (CFS) of patients [55]. Another prostate cancer cohort study stratified the patients according to the exclusive expression pattern of TFF3 and ERG. SPINK1 expression was observed exclusively in an aggressive subgroup of cancers that express TFF3, and SPINK1 was identified as a predictive biomarker for biochemical recurrence in univariate (*p* = 0.0009) and multivariate (*p* = 0.0003) analyses [56]. In colorectal cancer, a relatively high SPINK1 expression level correlated with a poor prognosis and a high Ki-67 labeling index [18,34]. Eighty human hepatocellular carcinoma patients were evaluated during a follow-up after curative resection. The patients with specimens displaying high SPINK1 levels were associated with unfavorable overall survival (*p* = 0.0001) and recurrence-free survival (*p* = 0.001) [57]. In contrast, a high SPINK1 expression level was associated with favorable overall survival in a Kaplan–Meier analysis of stage IV colon cancer patients receiving cetuximab-based targeted therapy. In particular, multivariable analysis further indicated an independent correlation with the hazard ratio (HR: 0.416; CI: 0.217 to 0.797; *p* = 0.008) [58].

*SPINK1* RNA expression profiles investigated by RNA-Seq and microarray platforms have been released together with the clinical follow-up patient data from public databases, including The Human Protein Atlas/The Pathology Atlas [31,59,60,61,62], SurvExpress [63], TCGA [46], and the Kaplan–Meier plotter database [64], which illustrate the prognostic value of *SPINK1* in specific cancer types (Table 1). *SPINK1* was a poor prognostic marker in cohorts of glioma, head and neck cancer, liver cancer, pancreatic cancer, renal cancer, and gastric cancer, while patients with colorectal cancer, urothelial cancer, and ovarian cancer expressing high *SPINK1* levels are associated with favorable outcomes.

## 5. SPINK1 and Cancer Cell Proliferation

The role of SPINK1 in modulating cancer cell proliferation has been reported. In lung adenocarcinoma, forced overexpression of SPINK1 increased PC9 and H1299 cell proliferation [48]. A similar effect was shown by tumor volume measurements in an animal model [48]. In non-small cell lung cancer, another subtype of lung cancer, functional analyses indicated that SPINK1 induced tumor cell growth and inhibited apoptosis by maintaining redox homeostasis driven by regulating the nuclear factor erythroid 2-related factor two pathways [45]. SPINK1 also augmented hepatocellular carcinoma cell proliferation ability in a CCK-8 cell proliferation assay [42]. In addition, increased SPINK1 expression and recombinant protein addition both induced cell proliferation in the BRL-3A rat liver hepatocyte line [10]. Lentiviral-based stable SPINK1 overexpression also increased the proliferative capacity of the AsPC-1 human pancreatic cancer cell line [65]. The increased pancreatic cancer cell proliferation by SPINK1 was further reported of which mechanism was through EGFR-mediated signaling activation [13]. In ovarian cancer, cancer cell proliferation was induced by SPINK1 and was further abolished by EGFR inhibitor [12]. In a colorectal cancer study, Spink3 (the mouse homolog of SPINK1) heterozygous mice showed a decrease in tumor volume compared with wild-type mice, in addition to induced cell proliferation observed in colon cancer cell lines [33]. The knockdown of SPINK1 expression by another study showed a significant decrease in the proliferation of colon adenocarcinoma WiDr cells, whereas the SPINK1-enriched conditioned medium increased the oncogenic phenotype [66]. Conditioned medium containing SPINK1 promoted prostate cancer PCa cell proliferation, and SPINK1 silencing reversed this phenotype [9].

## 6. SPINK1 and Cancer Metastasis

The clinical correlation of SPINK1 in predicting malignant phenotypes, including metastasis, was revealed. In primary prostate cancer, the SPINK1+/ERG+ phenotype was shown to be associated with a higher Gleason grade and aggressive subpopulation with a higher risk of lymph node metastases [67]. Although ETS gene fusions have been characterized in most prostate cancers, the pivotal molecular alterations in ETS-negative cancers remain obscure. SPINK1 expression was specifically detected in a subset of ETS rearrangement-negative cancers. The knockdown of SPINK1 in the ETS-negative prostate cancer cell line 22RV1 attenuated invasiveness [40]. The PC9 and H1299 lung cancer cell lines exhibited increased matrix metalloproteinase 12-based migration and invasion after ectopic SPINK1 overexpression, whereas the knockdown of SPINK1 inhibited biological functions [48]. Increases in cell invasion and migration were also detected in hepatocellular carcinoma cells [42]. In addition, both SPINK1 and IL-6-induced SPINK1 expression augmented cell motility by regulating STAT3 signaling in colorectal cancer [34]. Furthermore, another colorectal cancer study characterized the role of SPINK1 in promoting cancer metastasis using chicken chorioallantoic membrane assays, murine xenograft studies, and metastasis models [66]. SPINK1 in the conditioned medium also stimulated prostate cancer cell migration in wound healing and transwell assays [9]. Metastasis of human hepatocellular carcinoma was modulated and the epithelial-mesenchymal transition (EMT) was induced via the activation of the MEK/ERK (mitogen-activated protein kinase kinase/extracellular signal-regulated kinase) signaling pathway [57]. The addition of recombinant SPINK1 induced cancer cell invasion in several human adenoma and carcinoma cells of the colon and breast via phosphoinositide-3-kinase, protein kinase C and Rho-GTPases/Rho kinase-dependent pathways [68]. In a study enrolling 265 breast cancer patients, detection of serum SPINK1 was reported as a noninvasive strategy to accurately discriminate patients with metastatic breast cancer [69].

## 7. SPINK1 and Drug Resistance

Several studies have revealed the role of SPINK1 in drug resistance in cancer. SPINK1 is epigenetically repressed by miR-338-5p/miR-421. Overexpression of miR-338-5p/miR-421 reduced ABCG2 transporter-mediated Hoechst 33,342 efflux in a side population analysis in prostate cancer, suggesting the potential modulation of SPINK1 in drug resistance [36], a finding proven by direct SPINK1 silencing using specific shRNA clones [37]. Furthermore, the phenomenon was substantiated by another group showing the SPINK1-dependent increase in chemoresistance in the prostate cancer cell lines PC3, DU145, LNCaP and M12 treated with AG-1478 or cetuximab [9]. However, in contrast to these findings in colorectal cancer, PRSS expression levels were negatively correlated with the sensitivity of cancer cells following cetuximab treatment. SPINK1 was found to abolish the proteolytic cleavage of bevacizumab by recombinant PRSS1 in phosphate-buffered saline (PBS). Treatment with cetuximab or bevacizumab combined with SPINK1, a PRSS inhibitor, reduced tumor growth efficiently compared with cetuximab or bevacizumab alone in xenograft models [70]. The results suggest the dual role of SPINK1 in drug resistance.

## 8. SPINK1, Transdifferentiation, and Cancer Stemness

SPINK1 induces the epithelial-mesenchymal transition, cellular plasticity, and cancer stemness [36,37]. Neuroendocrine prostate cancer (NEPC) is an aggressive type of prostate cancer that can emerge following androgen-deprivation therapy (ADT). Importantly, increased SPINK1 and NEPC biomarkers were detected in the tumors of androgen receptor antagonist-treated mice and a subset of NEPC patients. Androgen deprivation was found to upregulate the expression of SPINK1 in neuroendocrine-transdifferentiated prostate cancer cells. The knockdown of SPINK1 elicited a decrease in sphere formation ability and the neuroendocrine phenotype, indicating that SPINK1 is critical in stemness maintenance [37]. The link between SPINK1 and increasing sphere formation ability in prostate cancer was also validated using the 22RV1 cell line and by regulating the expression of genes involved in stemness and epithelial-mesenchymal transition (EMT), including SNAI1 (SNAIL), SNAI2 (SLUG), and TWIST1 [36]. In addition, SPINK1 plays an important role in reprogramming the expression profile of prostate cancer cells and contributes to EET in prostate cancer [9].

## 9. Discussion and Conclusion

**Figure 4:**
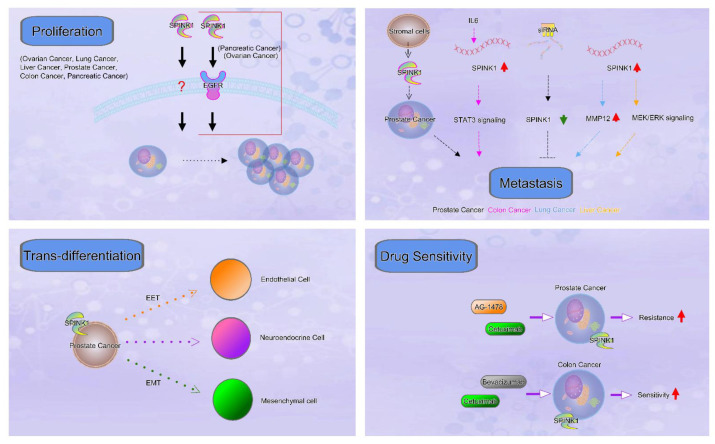
The representative scheme of SPINK1 in cancer progression was shown.

Recently published research results and data from several in silico analyses of the relative expression levels of SPINK1 in multiple types of cancers have been discussed in this review article. In addition to the clinical outcomes of patients displaying high SPINK1 levels on a pan-cancer scale, the pivotal roles of SPINK1 in regulating processes relevant to cancer progression, including cancer cell proliferation, metastasis, drug resistance, transdifferentiation, and cancer stemness, were summarized, and a representative scheme is shown (Figure 4). Notably, the differential expression of SPINK1 at the RNA level in specific cancer types further suggests the merits of mechanistic studies regarding alterations in transcriptional activity and RNA stability. In contrast, prognostic reports have revealed a discrepancy in several types of cancers, indicating that the impact of SPINK1 on those tumor types remains to be explored and that additional evidence is required. The prognostic power is influenced by the number of patients enrolled in each cohort. These differences may be due to the variations in analytic platforms and endpoint designs of the studies.

Aberrant SPINK1 that is secreted has been presumed to be a pathogenic factor. Recent observations indicated an uncommon mechanism that the several SPINK1 variants could lead to defective trypsin inhibition in chronic pancreatitis [71]. In addition, the K18Y mutant at the protease inhibitor site of SPINK1 was reported to inhibit tumor growth, angiogenesis, and the expression of several metastasis-related genes in colon cancer [68], suggesting the potential involvement of trypsin in cancer progression. Interestingly, the SPINK1-mediated function acting as a trypsin inhibitor was mostly not mentioned to be involved in the current cancer studies, also suggesting the merits of further exploring biological mechanisms of SPINK1 in regulating cancer progression. Investigations regard to identify novel interactive proteins could be considered. In addition, studies focusing on somatic mutational variants of the SPINK1 gene which elicit cancer-specific mechanisms in various types of cancers might shed light on the knowledge of SPINK1 in this field.

## Figures and Tables

**Table 1:** Correlation of *SPINK1* with cancer patient survival.

Symbol	Cancer Type	Prognosis	Endpoint	*p* Value	Case	Dataset	Method	Probe ID
*SPINK1*	Glioma	Poor	Overall survival	0.0045	153	TCGA	RNA Seq	
*SPINK1*	Thyroid Cancer	N/A	Overall survival	N/A	501	TCGA	RNA Seq	
*SPINK1*	Lung Cancer	-	Overall survival	N.S.	994	TCGA	RNA Seq	
*SPINK1*	Colorectal Cancer	Good	Overall survival	0.01	597	TCGA	RNA Seq	
*SPINK1*	Head and Neck Cancer	Poor	Overall survival	<0.001	499	TCGA	RNA Seq	
*SPINK1*	Stomach Cancer	-	Overall survival	N.S.	354	TCGA	RNA Seq	
*SPINK1*	Liver Cancer	Poor	Overall survival	0.008	365	TCGA	RNA Seq	
*SPINK1*	Pancreatic Cancer	Poor	Overall survival	0.038	176	TCGA	RNA Seq	
*SPINK1*	Renal Cancer	Poor	Overall survival	<0.001	877	TCGA	RNA Seq	
*SPINK1*	Urothelial Cancer	Good	Overall survival	<0.001	406	TCGA	RNA Seq	
*SPINK1*	Prostate Cancer	-	Overall survival	N.S.	494	TCGA	RNA Seq	
*SPINK1*	Testis Cancer	-	Overall survival	N.S.	134	TCGA	RNA Seq	
*SPINK1*	Breast cancer	-	Overall survival	N.S.	1075	TCGA	RNA Seq	
*SPINK1*	Cervical Cancer	-	Overall survival	N.S.	291	TCGA	RNA Seq	
*SPINK1*	Endometrial Cancer	-	Overall survival	N.S.	541	TCGA	RNA Seq	
*SPINK1*	Ovarian Cancer	-	Overall survival	N.S.	373	TCGA	RNA Seq	
*SPINK1*	Melanoma	-	Overall survival	N.S.	102	TCGA	RNA Seq	
*SPINK1*	Breast cancer	-	Relapse-free survival	N.S.	3951	E-MTAB-365, E-TABM-43, GSE: 11121, 12093,	Array	206239_s_at
						12276, 1456, 16391, 16446, 16716, 17705, 17907,		
						18728, 19615, 20194, 20271, 2034, 20685, 20711,		
						21653, 2603, 26971, 2990, 31448, 31519, 32646,		
						3494, 37946, 41998, 42568, 45255, 4611, 5327,		
						6532, 7390, 9195		
*SPINK1*	Ovarian cancer	Good	Progression-free survival	0.015	1435	GSE: 14764, 15622, 18520, 19829, 23554, 26193,	Array	206239_s_at
						26712, 27651, 30161, 3149, 51373, 63885, 65986,	RNA Seq	
						9891, TCGA (N = 565)		
*SPINK1*	Lung cancer	-	Post progression survival	N.S.	344	CAARRAY, GSE: 14814, 19188, 29013, 30219,	Array	206239_s_at
						31210, 3141, 31908, 37745, 43580, 4573, 50081,	RNA-Seq	
						8894, TCGA (N = 133)		
*SPINK1*	Gastric cancer	Poor	Post progression survival	0.032	498	GSE: 14210, 15459, 22377, 29272, 51105, 62254	Array	206239_s_at
Survival data were collected from databases: The Human Protein Atlas, TCGA, and Kaplan–Meier plotter.								
N.S.: no significance.								
N/A: not applicable.								

## Data Availability

Not applicable.
